# Microwaves as “Co-Catalysts” or as Substitute for Catalysts in Organophosphorus Chemistry

**DOI:** 10.3390/molecules26041196

**Published:** 2021-02-23

**Authors:** György Keglevich

**Affiliations:** Department of Organic Chemistry and Technology, Budapest University of Technology and Economics, 1521 Budapest, Hungary; keglevich.gyorgy@vbk.bme.hu

**Keywords:** microwave, organophosphorus chemistry, catalyst, catalyst- and solvent-free, green chemistry

## Abstract

The purpose of this review is to summarize the importance of microwave (MW) irradiation as a kind of catalyst in organophosphorus chemistry. Slow or reluctant reactions, such as the Diels-Alder cycloaddition or an inverse-Wittig type reaction, may be performed efficiently under MW irradiation. The direct esterification of phosphinic and phosphonic acids, which is practically impossible on conventional heating, may be realized under MW conditions. Ionic liquid additives may promote further esterifications. The opposite reaction, the hydrolysis of P-esters, has also relevance among the MW-assisted transformations. A typical case is when the catalysts are substituted by MWs, which is exemplified by the reduction of phosphine oxides, and by the Kabachnik–Fields condensation affording α-aminophosphonic derivatives. Finally, the Hirao P–C coupling reaction may serve as an example, when the catalyst may be simplified under MW conditions. All of the examples discussed fulfill the expectations of green chemistry.

The spread of the microwave (MW) technique opened a new chapter in organic chemistry in whole [1,2,3], and also in its specialized fields, such as heterocyclic [3,4,5] and organophosphorus chemistry [2,6]. MW irradiation makes possible more efficient syntheses in terms of reaction time, selectivity, and purity [1,2]. The role of MWs in organic syntheses provoked sharp disputes, however, today there is an agreement that thermal effects are responsible for the beneficial effect of MWs [7,8]. A widely accepted concept is that the efficiency of the MW irradiation is the consequence of the statistically occurring local overheating in the bulk of the mixture [9,10,11].

During our work, we laid the stress on green chemical aspects within organophosphorus chemistry [12]. This will be summarized in this review article. Besides developing efficient syntheses, we could enhance reluctant or slow reactions by applying the MW irradiation (1). It was another option to promote further certain MW-assisted reactions by performing them in the presence of an ionic liquid additive/catalyst (2). It is another possibility to substitute certain catalysts by MW irradiation (3). Last but not least, MWs may simplify catalytic systems/catalysts (4). In this paper, options (1)–(4) will be discussed in detail, placing the particular reactions into literature context.

## 1. Microwave Irradiation Allowing the Inverse-Wittig Type Reaction That Is Reluctant on Conventional Heating

**Scheme 1:**
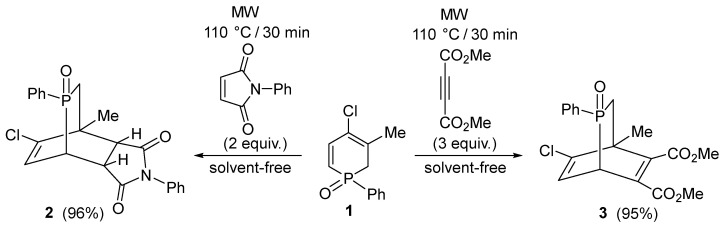
[4 + 2]Cycloadditions of a 1,2-dihydrophosphinine oxide (**1**) with dienophiles.

The Diels–Alder reaction of 1-phenyl-1,2-dihydrophosphinine oxide **1** with dienophiles, like *N*-phenylmaleimide and dimethyl acetylenedicarboxylate (DMAD), resulted in the formation of the respective phosphabicyclo[2.2.2]octene derivatives (**2**) and (**3**). The microwave (MW) technique was useful in shortening the reaction times and making the syntheses efficient. The MW-promoted reactions were 25 times faster than the thermal variations (Scheme 1) [13].

**Scheme 2:**
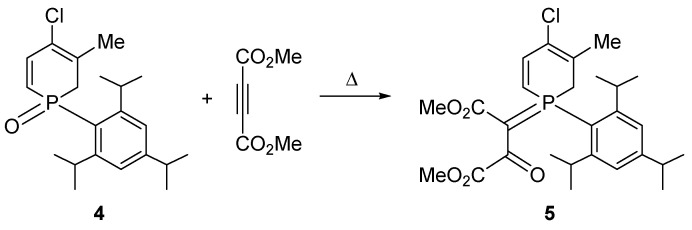
The inverse Wittig-type reaction of a P-aryl 1,2-dihydrohosphine oxide with dimethyl acetylenedicarboxylate (DMAD).

However, the 1-(2,4,6-triisopropylphenyl-1,2-dihydrophosphinine) oxide (**4**) [14] underwent an inverse-Wittig type transformation in reaction with DMAD to afford the corresponding β-oxophosphorane **5** (Scheme 2) [15,16].

**Scheme 3:**
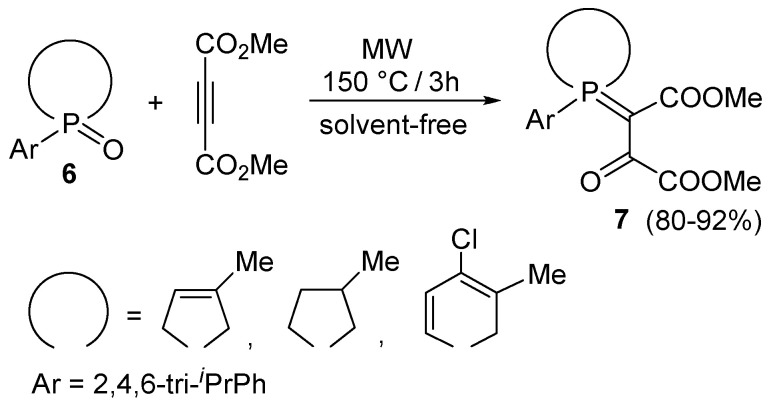
The inverse Wittig-type reaction of P-aryl ring phosphine oxides with DMAD.

Then, the new protocol seemed to be of a more general value [17]. The use of the MW technique was advantageous not only in the inverse Wittig-type reaction of 2,4,6-triisopropylphenyl-3-phospholene oxide but also in the reaction of 2,4,6-triisopropylphenylphospholane oxide and 2,4,6-triisopropylphenyl-1,2-dihydrophosphinine oxide (all represented by formula **6**) to afford β-oxophosphoranes **7** (Scheme 3) [18,19].

In the novel inverse Wittig-type reactions, MW irradiation made possible reactions at 150 °C for 3 h that were rather reluctant on conventional heating. Hence, MW acted as a kind of catalyst.

Theoretical calculations suggested a mechanism involving an oxaphosphete intermediate [20,21].

In the above case, the nature of the 1,2-dihydrophosphinine oxide determined its reactivity towards DMAD. Moreover, both the Diels–Alder cycloaddition and the inverse-Wittig type reaction took place efficiently on MW irradiation without the application of any catalyst.

## 2. Microwave-Assisted, Ionic Liquid-Catalyzed Direct Esterification of P-Oxoacids That Is Otherwise Impossible under Thermal Conditions

Phosphinic and phosphonic acids cannot be involved in direct esterification under common conditions. Only a few examples are known for the direct esterification of P-acids. These reactions required forcing conditions and were not efficient [22,23,24,25,26]. However, it was found by us that the P-acids can be esterified on MW irradiation.

**Scheme 4:**
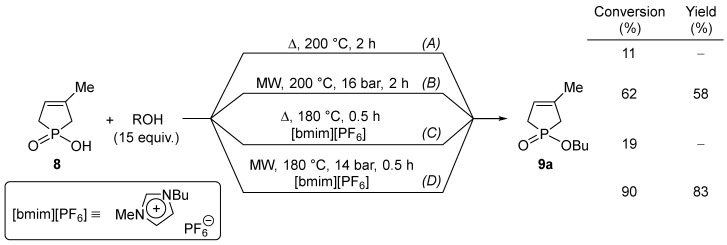
The MW-assisted catalytic direct esterification 1-hydroxy-3-methyl-3-phospholene 1-oxide (**8**).

The esterification of 1-hydroxy-3-phospholene oxide (**8**) with a series of alcohols at 180–235 °C afforded the alkyl phosphinates (**9**) in yields of 71–95% (Scheme 4/B vs. A, Table 1/Entries 1, 3, 5, 7, 9 and 11) [27,28,29]. The esterifications were less efficient with volatile and sterically hindered alcohols. The relatively high temperature required means a limitation that may be overcome by applying ionic liquids (ILs) as catalysts. It was found that in the presence of 10% of [bmim][PF_6_] as an additive, the esterifications took place at a lower temperature, and became faster and more efficient in shorter reaction times (Scheme 4/D, Table 1/Entries 2, 4, 6, 8, 10 and 12) [30]. The thermal direct esterifications were also somewhat promoted by the ionic liquid additive (Scheme 4/C).

Then, the MW-promoted IL-catalyzed direct esterifications were extended to other ring phosphinic acids, like 1-hydroxy-3,4-dimethyl-3-phospholene oxide (**10**), 1-hydroxy-phospholane oxides (**12** and **14**), as well as, a 1-hydroxy-1,2,3,4,5,6-hexahydrophosphinine oxide (**16**) (Table 2) [29,30,31].

Using IL-catalysis, even phenols could be the reactants in the MW-assisted esterification of cyclic phosphinic acids [32].

**Scheme 5:**
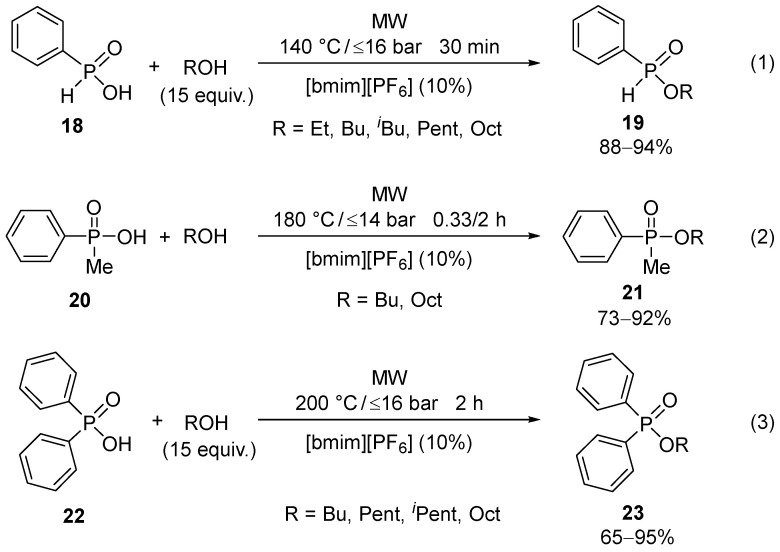
MW-promoted direct esterification of different phenyl-phosphinic acids (**18**, **20**, and **22**).

The esterification of the reactive phenyl-*H*-phosphinic acid (**18**) took place at a temperature of 160–190 °C to provide the phosphinates (**19**) in good (73–90%) yields [33]. The presence of [bmim][PF_6_] had a beneficial effect on the outcome (Scheme 5/(1)) [34]. Methyl-phenylphosphinic acid (**20**), and what is more, the sterically hindered diphenylphosphinic acid (**22**), could also be efficiently esterified in the presence of an IL (Scheme 5/(2) and (3)) [34,35].

It is noteworthy that thiobutanol could also be used under MW-assistance to afford the corresponding thiophosphinates [36]; however, the direct amidations of phosphinic acids were reluctant even on MW irradiation [37].

**Scheme 6:**
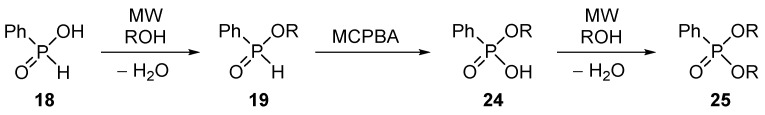
The preparation of dialkyl phenylphosphonates (**25**) via alkyl phenyl-*H*-phosphinates (**19**).

Our next targets were the phosphonic acids. In the first approach, the alkyl phenyl-*H*-phosphinates (**19**) were oxidized, then the ester-acid were esterified. However, we could not be satisfied with the efficiency (Scheme 6) [38].

For this, the direct esterification of phenylphosphonic acid was studied in detail. We learned that the MW-assisted and IL-catalyzed protocol furnished the monoesters (**27**) in good selectivities and in acceptable yields (Table 3). At the same time, the diesterification to species **25** was not efficient (Table 4). [Bmim][PF_6_] was somewhat less efficient than [bmim][BF_4_] [34,39].

As an alternative possibility, phenylphosphonic acid (**26**) was also subjected to alkylating esterifications using BuBr. These reactions were complete and selective for the diesterification only when BuBr was used in a 5-fold quantity at 120 °C (Table 5) [39].

At the same time, the monoalkyl phenylphosphonates (**27**) could be esterified further in reaction with alkyl halides under MW irradiation (Table 6) [39].

In this way, phenylphosphonates with (two) different alkyl groups (**28**) could also be prepared (Table 7). The isolated yields of products **28** were mostly around 70% [39].

**Scheme 7:**
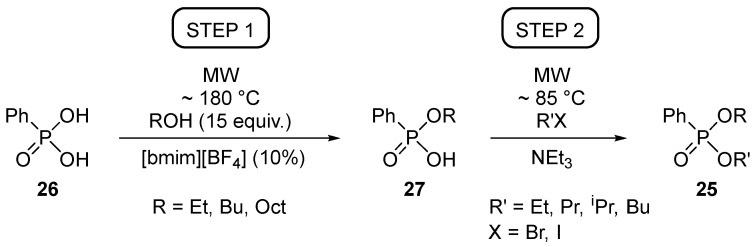
The new protocol elaborated for the synthesis of dialkyl phenylphosphonates.

It follows from our results that the method of choice for the diesterification of phosphonic acid **26** is when the first HO group of the phosphonic acid (**26**) is esterified with alcohol under MW conditions [34,39], while the second HO function (as in **27**) is converted to alkoxy by alkylation using an alkyl halogenide (Scheme 7) [39].

It is noteworthy that the MW-assisted continuous flow esterification of a *H*-phosphinic acid was also elaborated [40].

We were successful in modeling the rate-enhancing effect of MWs. The esterification of phenyl-*H*-phosphinic acid and 1-hydroxy-3-methyl-3-phospholene 1-oxide served as the model reactions [41,42].

**Scheme 8:**
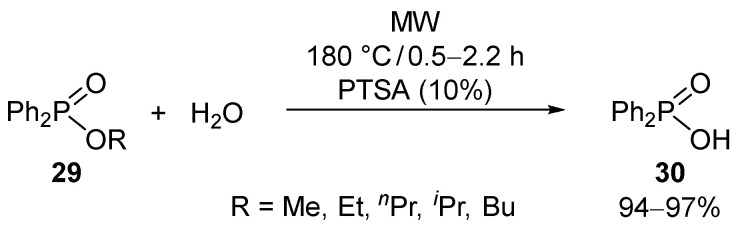
MW-assisted hydrolysis of alkyl diphenylphosphinates (**29**).

The opposite reaction of esterification is hydrolysis that is of high importance also in the sphere of P-esters [43,44]. The hydrolysis of phosphinates and phosphonates is carried out, in most cases, under acidic conditions [45,46,47,48,49,50,51,52], but, among other possibilities, base-catalyzed cases also occur [53,54,55,56,57]. It was a new approach to perform the hydrolyses under MW irradiation. Alkyl diphenylphosphinates (**29**) were hydrolyzed at 180 °C in the presence of 10% of *p*-toluenesulfonic acid (PTSA) as the catalyst (Scheme 8) [58]. For the esters with *n*-alkyl substituents, completion of the hydrolysis required 1.5–2.2 h; however, with the *i*-propyl ester, the hydrolysis took place after 0.5 h. This latter experience is due to the realization of the A_Al_1 mechanism. The acid (**30**) was isolated in yields of 94–97%.

A comparative thermal experiment afforded the phosphinic acid (**30**) in a lower conversion of 24%, indicating the beneficial effect of MWs. This is the consequence of the local overheating [27,28] and the better MW absorbing effect of PTSA.

In conclusion, MW irradiation made possible the otherwise impossible direct esterification of phosphinic acids, and the monoesterification of phosphonic acids. MW irradiation proved to be a useful tool in overcoming the enthalpy of activation barriers higher than 130 kJ mol^–1^ [27,28]. This is due to the beneficial effect of the statistically occurring local overheating [41,42]. On the other hand, the MWs were beneficial also in the acid-catalyzed hydrolysis of phosphinates.

## 3. Microwave as a Substitute for the Catalysts in the Deoxygenation of Phosphine Oxides

Besides the widely applied trichlorosilane (Cl_3_SiH) and phenylsilane (PhSiH_3_) [59,60], the use of the cheaper ethoxysilanes, (EtO)_3_SiH and (EtO)_2_MeSiH, as well as 1,1,3,3-tetramethyldisiloxane (TMDS) and polymethylhydrosiloxane, called also as methylpolysiloxane ([PMHS or MPS] represented by formula [–O–SiH(Me)–]_n_), offers alternative possibilities [61,62]. However, these silanes are of low reactivity. Beller et al. tested different acids as catalysts in the deoxygenation of triphenylphosphine oxide (**31**) with diethoxymethylsilane as the reductant (Table 8) [61]. No deoxygenation occurred in the lack of a catalyst. Adding 15 mol% of benzoic acid to the mixture, triphenylphosphine (**32**) was obtained in a yield of 6%. However, in the presence of the diphenyl ester of phosphoric acid as the catalyst, the yield of PPh_3_ was 75%. Moreover, it was observed that the use of a P-ester-acid catalyst with an electron-withdrawing substituent in the phenyl ring led to the quantitative reduction.

Different tertiary phosphine oxides (**33**) were reduced to the respective phosphines (**34**) using (EtO)_3_SiH in the presence of titanium(IV) isopropoxide as the catalyst (Table 9). The deoxygenations were performed in tetrahydrofuran (THF). On heating at 67 °C, the completion required 1 h, if the silane was applied in a 3-fold excess [62].

The reduction of aryl-diphenylphosphine oxides with (EtO)_3_SiH in the presence of Ti(O*^i^*Pr)_4_ in benzene at reflux provided the corresponding phosphine in 90% yield after a reaction time of 30 min [63].

The deoxygenation of triphenylphosphine oxide (**31**) was also elaborated using TMDS in the presence of catalysts. Copper(II) triflate was a suitable promoter in the reduction of the P=O unit at 100 °C in PhMe [64]. Without a catalyst, even measuring in TMDS in a 12 equivalents’ quantity, no reduction occurred. The use of 15 mol% of the (PhO)_2_P(O)OH in boiling PhMe also promoted deoxygenation [61]. Using 10 mol% of Ti(O*^i^*Pr)_4_ at 100 °C, triphenylphosphine (**32**) was formed in a conversion of 86%. In the presence of 1% of InBr_3_ as the catalyst, the reduction was quantitative at 100 °C [65] (Table 10).

It is noteworthy that there is no need for any catalyst if the reduction of triphenylphosphine oxide (**31**) with TMDS is performed in a solvent-free manner under MW irradiation. After treatment at 200 °C for 6.5 h, the reduction was quantitative. On conventional heating at 175 °C, there was a need for a 1-day reaction time. As the reduction takes place in the absence of catalyst, the MW-assisted approach may be regarded as a “green” protocol (Table 11) [66,67]. Practically, MW irradiation substituted the catalyst.

The reduction of ring phosphine oxides, such as 3-methyl-1-phenyl-2-phospholene oxide (**35a**), was performed using TMDS together with InBr_3_ as the catalyst in PhMe at 100 °C [65]. The deoxygenation also took place in the absence of catalyst in PhMe at reflux [68]. The solvent- and catalyst-free reduction of 1-phenyl-3-phospholene oxide **35b** was complete after a shorter reaction time in both the thermal and MW-promoted variations (Table 12) [66].

The next user-friendly silane, PMHS can be used in the deoxygenation of triphenylphosphine oxide (**31**). At 290 °C in the absence of any solvent, triphenylphosphine (**32**) was isolated in an 86% yield [69]. It is a disadvantage that the application of PMHS requires a relatively high temperature. Applying the reducing agent in a 12 equivalents’ quantity at 100 °C in PhMe, no reduction took place after 2 h. At the same time, applying Cu(OTf)_2_ as the promoter at 100 °C for 15 h, the deoxygenation took place [64]. Applying 15 mol% of (PhO)_2_P(O)OH as the catalyst at reflux for 1 day, the conversion was incomplete, and the phosphine (**32**) was obtained in a 35% yield [62]. Applying PMHS at a temperature of 175 °C on conventional heating in the absence of any catalyst and solvent, completion of the reduction required 17 h. In the MW-promoted version, 8 h was enough for an almost quantitative outcome (Table 13).

The first deoxygenation of 1-phenyl-2-phospholene oxide **35a** with PMHS was performed in the absence of any solvent at 250 °C [69]. Then, this transformation was carried out in PhMe at reflux 6 h [68]. The deoxygenation of 1-phenyl-3-phospholene oxide **35b** was also investigated under thermal and MW-promoted, in most cases, solvent-free conditions. These deoxygenations were complete at 110 °C after 4 and 2 h, respectively (Table 14) [66,67]. The outcome of the deoxygenation of the dimethylphospholene oxide **35c** was better in PhMe at reflux.

Optimum conditions for the reduction of 1-alkyl-3-methyl-3-phospholene 1-oxides by PhSiH_3_, TMDS, and PMHS were also evaluated [70].

TMDS and PMHS are user-friendly and low-cost reducing agents. The lower reactivity can be compensated by a solvent-free and MW-assisted protocol. MWs can be regarded as a kind of promoter substituting efficient catalysts.

## 4. Microwave as a Substitute for Catalysts in the Kabachnik–Fields Reaction

**Scheme 9:**
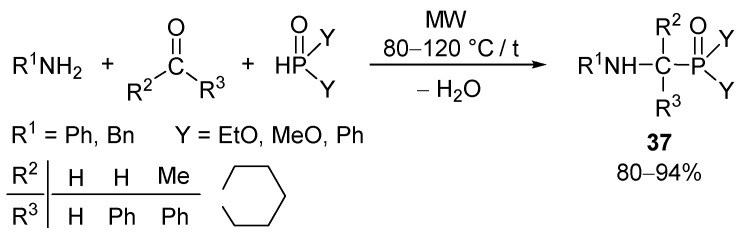
MW-assisted phospha-Mannich condensations.

α-Aminophosphonic acids, the P-analogues of α-amino acids, are of importance due to their potential biological activity, which is the consequence of their enzyme inhibitory properties [71]. The major method for the synthesis of α-aminophosphonates is the Kabachnik–Fields condensation of amines, aldehydes or ketones, and dialkyl phosphites [72,73]. α-Aminophosphonates (**37**, Y = RO) and α-aminophosphine oxides (**37**, Y = Ph) were synthesized by the solvent- and catalyst-free MW-assisted phospha-Mannich reaction of primary amines, oxo compounds, and dialkyl phosphites or diphenylphosphine oxide. Earlier preparations utilized special catalysts, such as, tetra-*tert*-butyl-substituted phthalocyanine—AlCl_3_ complex [74], magnesium perchlorate [75], metal triflates (M(OTf)_n_, M = Li, Mg, Al, Cu and Ce) [76], indium(III) triflate [77], bismuth(I) nitrate [78], samarium(II) iodide [79], ceric ammonium nitrate (CAN) [80], indium(III) chloride [81], and a variety of lanthanide (Yb, Sm, Sc, La) triflates [82], which mean cost and environmental burden. It was found that under MW conditions, there is no need for any catalyst (Scheme 9) [83].

Starting from heterocyclic amines: pyrrolidine, piperidine, morpholine and piperazine derivatives or heterocyclic >P(O)H species, *N*-heterocyclic [84] and *P*-heterocyclic [85] α-aminophosphonates were obtained. 3-Amino-6-methyl-2*H*-pyran-2-ones were also suitable amino derivatives in Kabachnik–Fields reaction with formaldehyde and dialkyl phosphites or diphenylphosphine oxide [86]. As special cases, α-aminophosphonates with different alkoxy groups [87], α-aminophosphinates [88], and α-aminophoshonates with sterically demanding α-aryl substituents [89] were also synthesized under MW-assisted conditions. Moreover, carboxylic amides could also be used under solvolytic conditions [90]. It is noteworthy that the phospha-Mannich reactions may also be performed in the presence of the T3P^®^ activating agent [91].

**Scheme 10:**
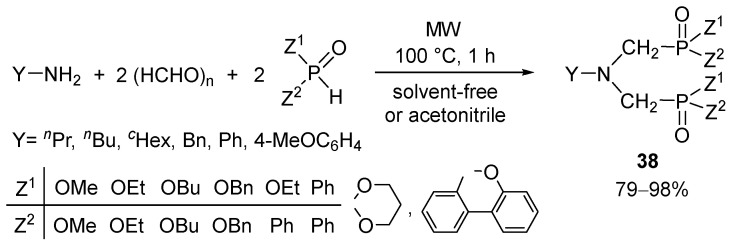
The bis(phospha-Mannich) reaction.

Primary amines are suitable components for bis(Kabachnik–Fields) condensations [92]. In these distances, alkyl or arylamines were reacted with two equivalents of the formaldehyde and the >P(O)H reagents to afford the bis(Z^1^Z^2^P(O)CH_2_)amines (**38**) (Scheme 10) [93,94,95]. Most of the reactions could be carried out in a solvent-free manner.

The bisphosphinoyl derivatives (**38**, Z^1^ = Z^2^ = Ph) were transformed after double-deoxygenation to bis(phosphines) that were useful in the synthesis of ring platinum complexes [94,95,96] α-, β- and γ-amino acids (or esters) were also utilized in the double Kabachnik–Fields condensation to furnish the bis(phosphono- or phosphinoyl) products [97,98].

The α-aminophosphonates may be formed via imine of α-hydroxyphosphonate intermediates [72,92,99,100]. α-Hydroxyphosphonates may be formed in a reversible manner from the corresponding oxo compound and dialkyl phosphite [101,102]. It was a somewhat surprising experience that the α-hydroxyphosphonates could be converted to the respective α-aminophosphonates by reaction with primary amines under MW conditions. This was promoted by a favorable adjacent group effect [103,104].

In summary, a wide range of mono- and bis Kabachnik–Fields reactions were carried out under MW-assisted and catalyst-free conditions, and mostly in a solvent-free manner.

**Scheme 11:**
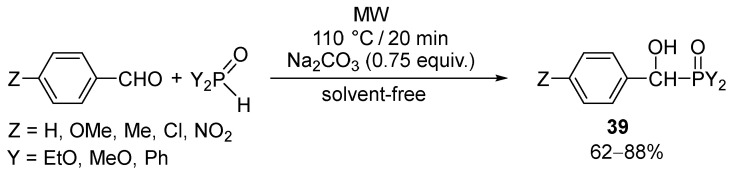
MW-promoted synthesis of α-hydroxyphosphonates and α-hydroxyphosphine oxides by the Pudovik reaction.

α-Aryl-α-hydroxyphosponates [105] mentioned above as intermediates in the phospha-Mannich condensations, along with α-aryl-α-hydroxyphosphine oxides (**39**), were synthesized in a catalytic and solvent-free MW-assisted Pudovik reaction comprising the addition of >P(O)H species to aryl aldehydes (Scheme 11) [106].

Dialkyl phosphites could also be reacted with α-ketophosphonates to result in the formation of dronate analogue α-hydroxybisphosphonates in the presence of diethylamine and in the absence of any solvent [107,108].

It was found that the Pudovik reaction may also be realized at room temperature in a solvent-free manner. However, these methods required special catalysts, such as piperazine [109], magnesium chloride/3 equivalents of triehylamine [110], barium hydroxide [111], sodium carbonate [112], potassium phosphate [113], sodium-modified fluoroapatite [114], and silica-supported tungstic acid [115].

Moreover, the work-up needed a considerable quantity of solvents. Hence, these methods cannot be regarded as green. The author of this review together with co-workers developed an indeed environmentally-friendly method by crystallizing the products from the mixtures [116].

## 5. Microwave Irradiation Allowing the Simplification of the Catalysts in the Hirao Reaction

**Scheme 12:**
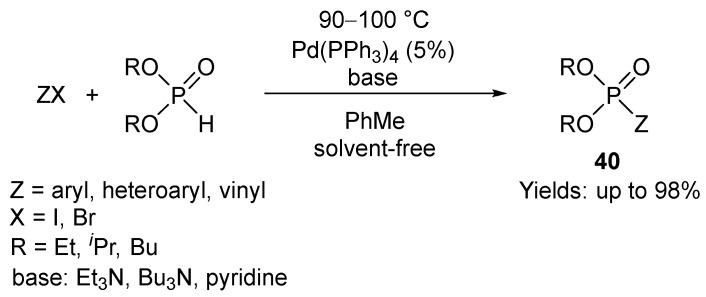
The classical P–C coupling reactions.

The Hirao reaction of aryl, heteroaryl, and vinyl halogenides (or other derivatives) with dialkyl phosphites, alkyl *H*-phosphinates, and secondary phosphine oxides is an important method for the synthesis of phosphonates, phosphinates, and phosphine oxides, respectively [117,118,119]. The original Hirao P–C coupling aimed at the synthesis of arylphosphonates reacting aryl- and vinyl halogenides with dialkyl phosphites in the presence of tetrakis(triphenylphosphine)palladium (Scheme 12) [120,121,122]. The P–C coupling reaction was then extended to other substrates [123,124,125,126,127,128,129,130,131,132,133,134,135] that was followed by further variations involving different >P(O)H reagents, Pd(II)-, or other metal (Ni(II) and Cu(II)) salts as catalyst precursors together with mono- and bidentate P-ligands, bases (mostly amines) and solvents.

Instead of Pd(PPh_3_)_4_, the application of Pd salts together with *P*-ligands was spread. In such cases, the Pd(0) catalyst is formed in situ from the components. From among the Pd salts, Pd(OAc)_2_ is the most suitable. The Pd(OAc)_2_/P-ligand combination was often utilized in the preparation of arylphosphonates, and this approach was more suitable than the classical Pd(PPh_3_)_4_ catalyst [136,137,138,139,140,141,142,143,144,145,146,147,148]. PPh_3_, dppp, dppb, dppe, dppf, and BINAP (2,2′-bis(diphenylphosphino)-1,1′-binaphthyl were the typical P-ligands applied.

**Scheme 13:**
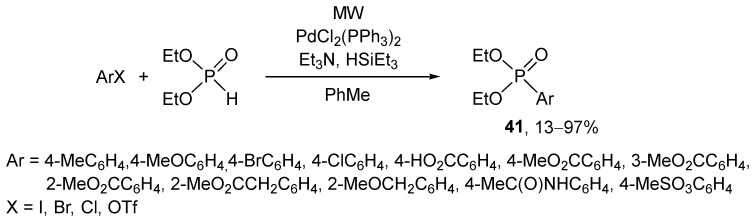
The first P–C coupling realized on MW irradiation.

“Greener” and more efficient protocols were developed for the Hirao reaction in the last twenty years. The MW technique also proved to be useful in the Hirao reactions. The first MW-assisted P–C coupling took place between aryl halides/triflates and diethyl phosphite in the presence of bis(triphenylphosphine)palladium dichloride, triethylamine, and triethylsilane as the reductant (Scheme 13) [149]. The Hirao reaction was performed in a domestic microwave device.

**Scheme 14:**
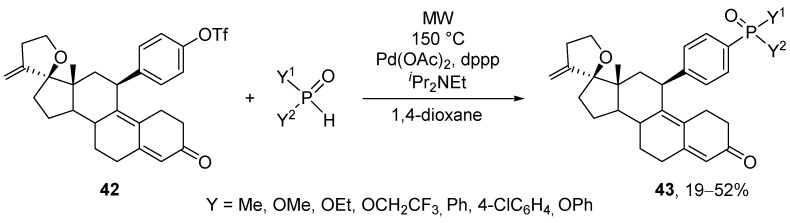
The derivation of 11β-aryl-substituted steroids with the help of the Hirao reaction.

The MW-assisted Hirao reaction of dialkyl phosphites with aryl and vinyl halides/triflates was also studied in the presence of Pd(PPh_3_)_4_ [150]. The best results (72–96%) were obtained using Cs_2_CO_3_ in THF. The MW protocol was also applied in the synthesis of P-functionalized 11β-aryl-substituted steroids (**43**) that are progesterone receptor antagonists (Scheme 14) [151].

**Scheme 15:**
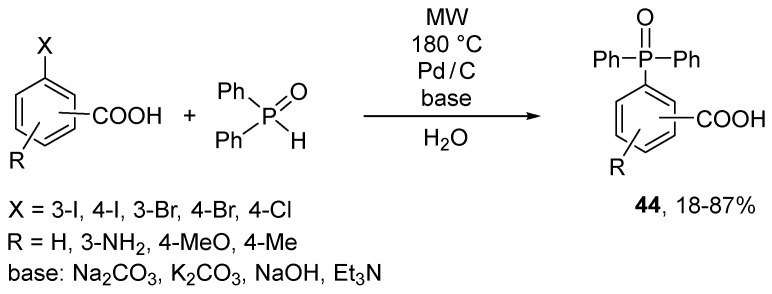
Hirao reaction in water using Pd/C on MW irradiation.

A few arylboronic acids and arylfluoroborates were coupled with dialkyl phosphites using the Pd(OAc)_2_ or Pd(O_2_CCF_3_)_2_/dmphen catalyst combination, and *p*-benzoquinone in the absence of a base [152]. The authors assumed the role of the reoxidant in the catalytic cycle. The author of this review believes that the application of an oxidant in the P–C coupling is mistaken. Instead, a reductive agent may be useful. A new cyclodiphosphazane-containing Pd catalyst was tested in the preparation of triarylphosphine oxides from aril bromides and diphenylphosphine oxide [153]. The use of this exotic promoter and Cs_2_CO_3_ as a base in acetonitrile under MW irradiation gave Ph_3_P=O in yields of 46–95%. It is noteworthy that the coupling of iodo- and bromobenzoic acids with diphenylphosphine oxide could be performed in water using Pd/C catalyst under MW conditions (Scheme 15) [154]. In this case, the tetrabutylammonium bromide additive had no influence on the outcome.

**Scheme 16:**
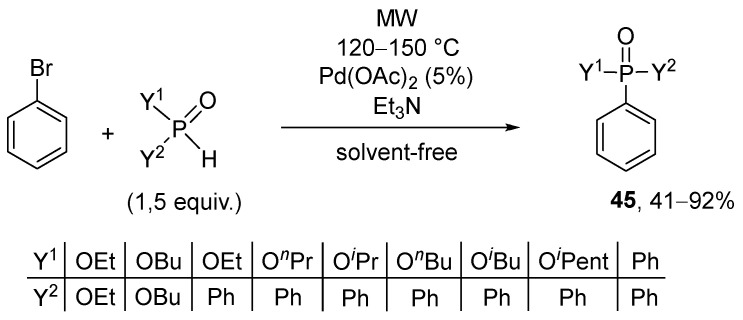
MW-promoted P–C couplings applying Pd(OAc)_2_ without added P-ligands.

It was recognized by the *Keglevich* group that the Hirao cross-couplings may take place in the presence of Pd(OAc)_2_ without the addition of the usual P-ligands under solvent-free and MW-assisted conditions (Scheme 16) [155]. The reaction of bromobenzene and 1.5 equivalents of the dialkyl phosphites, phenyl-*H*-phosphinates, and diphenylphosphine oxide was carried out in the presence of 5% of Pd(OAc)_2_ and 1.1 equivalents of triethylamine. The relevance of the MW technique was pointed out by comparative thermal experiments. The conversion was almost complete at 120 °C, but the best results were obtained at 150 °C.

In the next stage, the Pd(OAc)_2_-promoted “P-ligand-free” Hirao reactions were extended to different bromoarenes (Table 15) [156]. The experience was that both electron-releasing and electron-withdrawing substituents decreased the reactivity, as in these instances, higher temperatures (175–200 °C) were necessary to obtain the aryl phosphonates in acceptable yields (69–92%) (Table 15). The reactions of the methoxy- and alkyl-substituted bromoarenes with a decreased reactivity required, in most cases, a temperature of 200 °C, and the application of 10% of the Pd(OAc)_2_ catalyst.

**Scheme 17:**
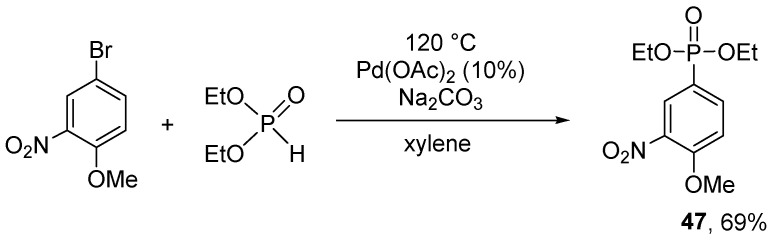
An additional Pd(OAc)_2_-catalyzed “P-ligand-free” P–C coupling.

Recently, Hirao et al. have publised another “P-ligand-free” reaction (Scheme 17) [157]. According to this, diethyl phosphite was coupled with 2-nitro-5-bromoanisole applying Pd(OAc)_2_ as the catalyst and Na_2_CO_3_ in xylene at 120 °C to furnish the respective aryl phosphonate (**47**) in a yield of 69% after 24 h.

Xiao and his co-workers described the Pd-catalyzed cross-coupling of an arylsulfinate salt with dialkyl phosphites applying PdCl_2_ without the usual P-ligands in DMF–DMSO [158]. The arylphosphonates were prepared in good yields using silver carbonate as the oxidant under MW irradiation. Here, it is noted again that there is no need for an oxidant during the P–C coupling. The Pd(OAc)_2_-promoted “P-ligand-free” protocol was extended to the Hirao reaction of heteroaryl bromides [159], and the reactivity of the substrates was studied in detail [160].

Moreover, the mechanism of the Pd-catalyzed Hirao reactions carried out using the P-reactant in excess was investigated experimentally and by quantum chemical calculations [161]. It was found that if Pd(OAc)_2_ is applied in a quantity of 10%, the >P(O)H reactant should be used in a quantity of 1.3 equivalents. 10% of the P-species reduces Pd(II) to Pd(0), while 20% covers the P-ligand that is the trivalent tautomeric form (>P–OH) of the >P(O)H reagent. The whole catalytic cycle involving oxidative addition, ligand exchange, and reductive elimination was adapted to our model, and the elemental steps were refined [161]. The formation of the “PdP_2_” catalyst and its activity were investigated under a separate cover [162]. It turned out that the Ar_2_POH ligands with 2-MePh or 3,5-diMePh substituents are more advantageous than the Ph one, as the steric hindrance prevents the tricoordination of the Pd [162].

**Scheme 18:**
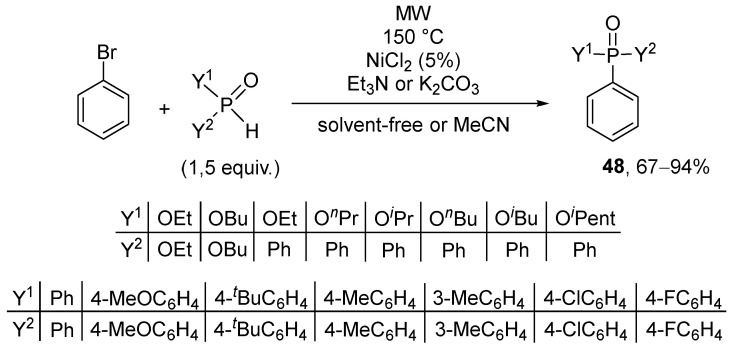
Hirao reactions applying NiCl_2_ as the catalyst without added P-ligands.

It was also found that NiCl_2_ may also be a suitable catalyst in the P–C coupling of bromobenzene and a series of >P(O)H reagents (Scheme 18) [163]. The experiments were carried out at 150 °C on MW irradiation. Using 1.5 equivalents of NEt_3_ in a solvent-free manner, completion of the reaction of diethyl phosphite and bromobenzene required 2 h, and the diethyl phenylphosphonate was isolated in a 67% yield. The use of K_2_CO_3_ in acetonitrile was more advantageous: in the presence of 5% NiCl_2_, after a reaction time of 45 min the yield of the corresponding product was 92%. Applying phenyl-*H*-phosphinates, the diphenylphosphinates were isolated in yields of 84–89%. Diphenylphosphine oxide and other aryl-substituted secondary phosphine oxides served as additional reagents in the “P-ligand-free” P–C couplings under discussion.

**Scheme 19:**
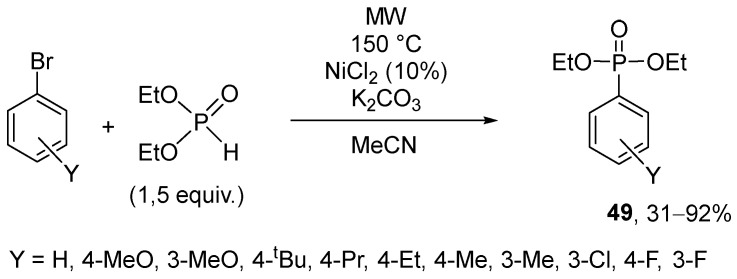
NiCl_2_-catalyzed P–C couplings of bromoarenes with diethyl phosphite.

The NiCl_2_-catalyzed phosphonylation of a series of bromoarenes gave similar results as those in the presence of Pd(OAc)_2_. However, the scope of the aryl bromides was more limited (Scheme 19) [163].

The nature of the Ni-catalyst, its formation, and the mechanism of the NiCl_2_-catalyzed P–C coupling reactions was also studied experimentally and by theoretical calculations [163]. It was found that in these cases, a Ni(II)(PY_2_OH)_2_ type catalyst is formed, and Ni(II) is converted to Ni(IV) in the oxidative addition step. This surprising finding was also proved to be true for earlier Ni-catalyzed instances [164] carried out originally in the presence of Zn or Mg reductants [165,166,167]. Hence, the Ni(II) → Ni(IV) conversion may be of more general value instead of the earlier assumed Ni(0) → Ni(II) protocol [168].

Both the Pd- and the Ni-catalyzed protocols elaborated by the *Keglevich* group are suitable for the coupling of bromoarenes and different >P(O)H reagents. Considering the conditions, costs, and toxicity, one can conclude that the application of Pd(OAc)_2_ is more attractive, but the use of NiCl_2_ may be a good alternative as well. The recent developments and extensions of the P–C coupling reactions open a new horizon since there is no need to add sensitive and expensive P-ligands.

**Scheme 20:**
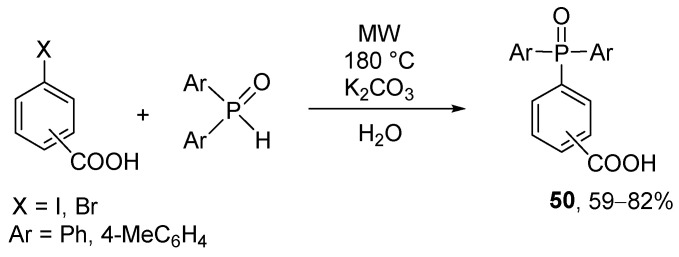
A catalyst-free Hirao reaction in aqueous medium.

A catalyst-free method was developed for the P–C coupling of halobezoic acids and secondary phosphine oxides in water as the medium (Scheme 20) [169]. 4-Iodo-, 3-bromo-, and 4-bromobenzoic acids were coupled with diaryl phosphine oxides under MW conditions at 180 °C for 1–6 h in the presence of K_2_CO_3_. The only limitation of this “green” P–C coupling reaction is the low water-solubility of the P-reagents.

In summary, the Hirao reaction utilizing a series of suitably substituted aryl derivatives and different >P(O)H reagents along with a Pd or Ni catalyst provides arylphosphonates, tertiary phosphine oxides, and related compounds that may be useful intermediates in synthetic organic chemistry. The chemistry discussed hides interesting green chemical aspects, such as MW activation, solvent- and catalyst-free protocols, as well as mechanistic delicates.

## Figures and Tables

**Table 1:**
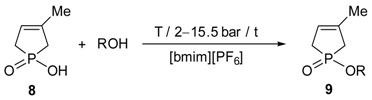
Esterification with other alcohols.

Entry	R	[bmim][PF_6_]	T (°C)	t (h)	Conversion (%)	Yield (%)
1	Pr	–	180	4	40	30
2	Pr	10%	180	3	98	68
3	Pent	–	220	2.5	100	82
4	Pent	10%	180	0.5	100	94
5	*^i^*Pent	–	235	3	100 *	76
6	*^i^*Pent	10%	180	0.5	100	95
7	Oct	–	220	2	95 *	71
8	Oct	10%	180	0.33	100	85
9	*^i^*Oct	–	220	1	100	76
10	*^i^*Oct	10%	180	0.33	100	84
11	Dodecyl	–	230	2	100	95
12	Dodecyl	10%	180	0.33	100	94

* Estimated value.

**Table 2:** Extension of the MW-assisted IL-catalyzed direct esterification to other cyclic phosphinic acids.

Model Reaction	[bmim][PF_6_]	T (°C)	t (h)	Yield (%)
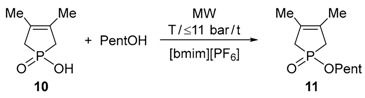	–	235	3	67
	10%	200	1	72
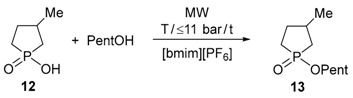	–	235	3	79
	10%	220	1	89
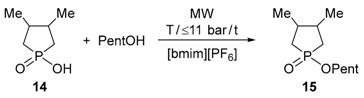	–	235	5	60
	10%	220	2	84
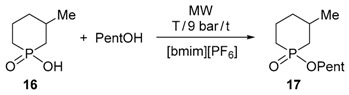	–	220	4	31
	10%	200	2	42

**Table 3:**
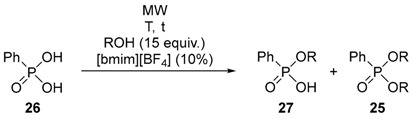
MW-promoted direct esterification of phenylphosphonic acid (**26**).

R	T (°C)	t (h)	Conversion (%)	Composition		Yield of the Monoester (%)
				Monoester (%)	Diester (%)	
Bu	180	0.75	100	95	5	82
Et	165	8	82	94	6	70
Oct	180	0.75	100	96	4	90

**Table 4:**
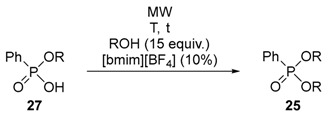
MW-promoted direct esterification of phenylphosphonic monoesters (**27**).

R	T (°C)	t (h)	Conversion (%)
Bu	220	6	45
Oct	235	3	72

**Table 5:**
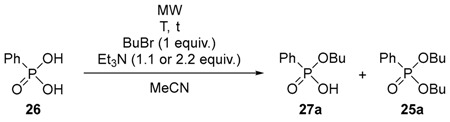
Alkylating esterification of phenylphosphonic acid (**26**).

BuBr (Equiv.)	T (°C)	t (h)	Conversion (%)	Composition	
				Monoester (%)	Diester (%)
1	100	2	62	61	39
2.2	100	4	95	32	68
5	120	4	100	17	83 *

* The diester was prepared in a yield of 69%.

**Table 6:**
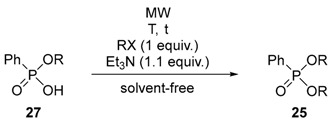
Alkylation of phenylphosphonic monoesters (**27**)—synthesis of dialkyl phenylphosphonates (**25**).

R	RX	T (°C)	t (h)	Yield (%)
Bu	BuBr	85 *	0.5	80
Et	EtI	85	0.5	92
Oct	OctBr	100	1	72

* The yield of the comparative thermal experiment was 57%.

**Table 7:**
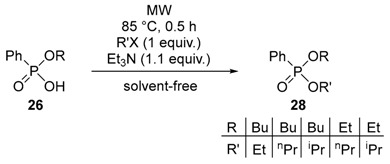
The preparation of phenylphosphonates (**28**) with different alkyl groups.

R	R’X	Yield of the “Mixed” Ester (%)
Bu	EtI	71
Bu	*^n^*PrBr	68
Bu	*^i^*PrBr	48
Et	*^n^*PrBr	72
Et	*^i^*PrBr	54
Et	BuBr	72

**Table 8:**
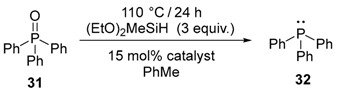
Deoxygenation of triphenylphosphine oxide (**31**) using (EtO)_2_MeSiH with acid catalysts.

Catalyst	Yield (%)
–	<1
PhCOOH	6
(PhO)_2_P(O)OH	75
(4-NO_2_C_6_H_4_O)_2_P(O)OH	>99
(4-CF_3_C_6_H_4_O)_2_P(O)OH	>99

**Table 9:**
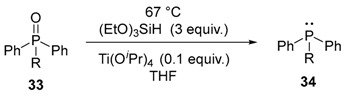
Reductions with triethoxysilane.

R	Yield (%)
Ph	85
CH_2_CH_2_P(O)Ph_2_	90
Me	99
Et	95
*^i^*Pr	81
*^i^*Pr	41
Bn	98
CH_2_Bn	83

**Table 10:**
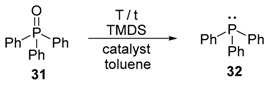
The catalytic deoxygenation of triphenylphosphine oxide (**31**) applying 1,1,3,3-tetramethyldisiloxane (TMDS) as the reducing agent.

Equivalent of TMDS	Catalyst (mol%)	T (°C)	t (h)	Conversion (%)
12	–	100	2	<1
3	Cu(OTf)_2_ (10)	100	15	96
3	(PhO)_2_P(O)OH (15)	110	24	62
1.2	Ti(O*^i^*Pr)_4_ (10)	100	24	86
3	InBr_3_ (1)	100	18	>99

**Table 11:**
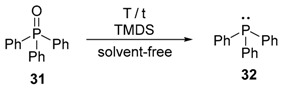
Deoxygenation of triphenylphosphine oxide (**31**) using TMDS in the absence of catalysts.

Equivalent of TMDS	Mode of Heating	T (°C)	t (h)	Conversion (%)
2	Δ	175	24	92
2	MW	200	6.5	100

**Table 12:**
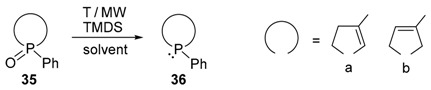
Deoxygenation of phospholene oxides (**35**) applying TMDS.

P=O	Equivalent of TMDS	Catalyst (mol%)	Solvent	Mode of Heating	T (°C)	t (h)	Conversion (%)
**a**	3	InBr_3_ (1)	PhMe	Δ	100	40	95
**a**	2	–	PhMe	Δ	110	8	82
**b**	2	–	–	Δ	110	5	100
**b**	2	–	–	MW	110	3	100

**Table 13:**
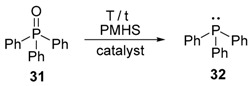
Deoxygenation of triphenylphosphine oxide with polymethylhydrosiloxane (PMHS).

Equivalent of PMHS	Catalyst (mol%)	Mode of Heating	Solvent	T (°C)	t (h)	Yield (%)
5	–	Δ	–	290	2	86
12	–	Δ	PhMe	100	2	0
6	Cu(OTf)_2_ (10)	Δ	PhMe	100	15	88
4	(PhO)_2_P(O)OH (15)	Δ	PhMe	110	24	35
2	–	Δ	–	175	17	87
2	–	MW	–	175	8	90

**Table 14:**
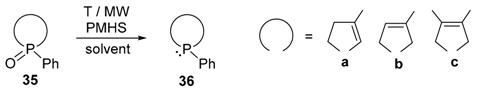
The deoxygenation of a series of phospholene oxides using PMHS.

Phosphine Oxide	Silane (Equiv.)	Mode of Heating	Solvent	T (°C)	t (h)	Yield (%)
**a**	5	Δ	–	250	2	88
**a**	2	Δ	PhMe	110	6	85
**b**	2	Δ	–	110	4	91
**b**	2	MW	–	110	2	92
**c**	5	Δ	–	250	–	35
**c**	2	Δ	PhMe	110	6	92

**Table 15:**
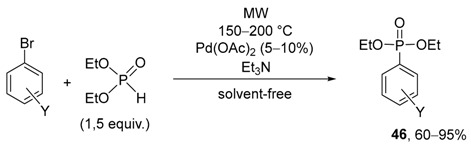
MW-assisted Hirao reaction of bromoarenes and diethyl phosphite applying Pd(OAc)_2_ as the catalyst precursor.

Y	Pd(OAc)_2_ (%)	T (°C)	t (min)	Conversion (%)	Yield (46) (%)
H	5	150	5	99	93
4-MeO	10	200	2	80	69
3-MeO	10	200	2	93	79
4-Pr	10	200	2	86	71
4-Et	10	175	15	93	85
4-Me	10	175	10	86	73
4-Cl	10	175	10	95	83
3-Cl	10	175	10	95	87
4-F	5	175	5	99	91
3-F	5	175	10	100	88
4-CO_2_Et	5	175	15	100	89
3-CO_2_Et	10	200	2	93	81
4-C(O)Me	5	175	5	96	71
3-C(O)Me	10	175	5	100	92

## Data Availability

The data presented in this study are available in the references.
